# The assessment of inter-individual variation of whole-genome DNA sequence in 32 cows

**DOI:** 10.1007/s00335-015-9606-7

**Published:** 2015-10-16

**Authors:** Joanna Szyda, Magdalena Frąszczak, Magda Mielczarek, Riccardo Giannico, Giulietta Minozzi, Ezequiel L. Nicolazzi, Stanislaw Kamiński, Katarzyna Wojdak-Maksymiec

**Affiliations:** Biostatistics Group, Department of Genetics, Wroclaw University of Environmental and Life Sciences, Kozuchowska 7, 51-631 Wroclaw, Poland; Fondazione Parco Tecnologico Padano, Via Einstein Albert, 26900 Lodi, LO Italy; DIVET, Università di Milano, Via Celoria 10, 20133 Milan, Italy; Institute of Animal Genetics, University of Warmia and Mazury, Oczapowskiego 2, Olsztyn, 10-719 Poland; West Pomeranian University of Technology, Piastów 17, 70-310 Szczecin, Poland

## Abstract

Despite the growing number of sequenced bovine genomes, the knowledge of the population-wide variation of sequences remains limited. In many studies, statistical methodology was not applied in order to relate findings in the sequenced samples to a population-wide level. Our goal was to assess the population-wide variation in DNA sequence based on whole-genome sequences of 32 Holstein–Friesian cows. The number of SNPs significantly varied across individuals. The number of identified SNPs increased with coverage, following a logarithmic curve. A total of 15,272,427 SNPs were identified, 99.16 % of them being bi-allelic. Missense SNPs were classified into three categories based on their genomic location: housekeeping genes, genes undergoing strong selection, and genes neutral to selection. The number of missense SNPs was significantly higher within genes neutral to selection than in the other two categories. The number of variants located within 3′UTR and 5′UTR regions was also significantly different across gene families. Moreover, the number of insertions and deletions differed significantly among cows varying between 261,712 and 330,103 insertions and from 271,398 to 343,649 deletions. Results not only demonstrate inter-individual variation in the number of SNPs and indels but also show that the number of missense SNPs differs across genes representing different functional backgrounds.

## Introduction

Despite the fact that a continuously growing number of whole-genome sequences are available for cattle, the knowledge of the bovine genome is still limited (Kõks et al. 2014). Up to now, most of the published results were based on the analysis of genomes of single (Eck et al. 2009; Kõks et al. 2013, 2014) or only a few individuals (Larkin et al. 2012; Lee et al. 2013; Stothard et al. 2011). Recently, a few studies in which a larger number of animals were sequenced (Baes et al. 2014; Brøndum et al. 2014; Daetwyler et al. 2014; Höglund et al. 2015; Jansen et al. 2013) were published. However, they did not apply statistical methodology in order to relate findings on single-nucleotide variation in the sequenced samples to a population-wide level. Such results are largely of a descriptive nature, which mainly describe individual characteristics of the sequenced animals. Moreover, without formal hypotheses testing, conclusions from analyses based on single or a few animals are highly prone to sampling and technological bias and, thus, may not be suitable for population-based conclusions. Daetwyler et al. (2014) and Höglund et al. (2015) used sequence information from a larger number of animals to perform imputation of whole-genome polymorphic variants in order to relate the variation in DNA sequence to the variation of (pseudo)phenotypes. Studies of Brøndum et al. (2014) and Daetwyler et al. (2014) were concentrated on the accuracy of imputation techniques, while Baes et al. (2014) compared different variant calling approaches. Since the focus of the aforementioned analyses was not on DNA variation itself, our study aims to extend the scope of a whole-genome sequence analysis of dairy cattle by considering the variation of single-nucleotide polymorphisms. The availability of whole-genome sequences for 32 individuals allows the incorporation of statistical testing procedures and consequently enables population-wide inferences.

## Materials and methods

### Animals

Whole-genome DNA sequences were available for 32 Polish Holstein–Friesian cows. These individuals were selected from a group of 991 case–control cows with clinical mastitis cases diagnosed by a veterinarian and their healthy herdmates. The experimental design comprised 16 paternal half-sib pairs matched according to the number of parities, production level, and birth year but differing in terms of their mastitis resistance expressed by the frequency of clinical mastitis diagnosed throughout their production life. More specifically, in each pair, one of the half-sibs represents an animal without clinical mastitis incidence throughout the whole production period and the other with multiple clinical mastitis cases.

### Whole-genome DNA sequencing

DNA was isolated from blood samples using a DNA Isolation System. The quality of the DNA was verified using a 2200 TapeStation DNA Screen Tape device and its concentration ascertained using fluorescence methods (Picogreen, LifeTechnologies). Libraries were generated from 1 ug of genomic DNA using the Illumina TruseqDNA PCR free sample prep kit following the manufacturer’s protocol and their evaluation was made with the Agilent Tape Station 2200. Fragments were quantified by Picogreen and then normalized to 10 nM as recommended by Illumina for clusters generation on the Hiseq2000. Libraries were denaturated and samples were run in a total of 32 lanes of Hiseq Flowcell. The Illumina Truseq PE cluster kit v3 was used to generate clusters on the grafted Illumina Flowcell and the hybridized libraries were sequenced on the Hiseq2000 with a 100 cycles of paired-end sequencing module using the Truseq SBS kit v3. All of the samples were sequenced on an IlluminaHiSeq2000 next-generation sequencing platform.

**Fig. 1:**
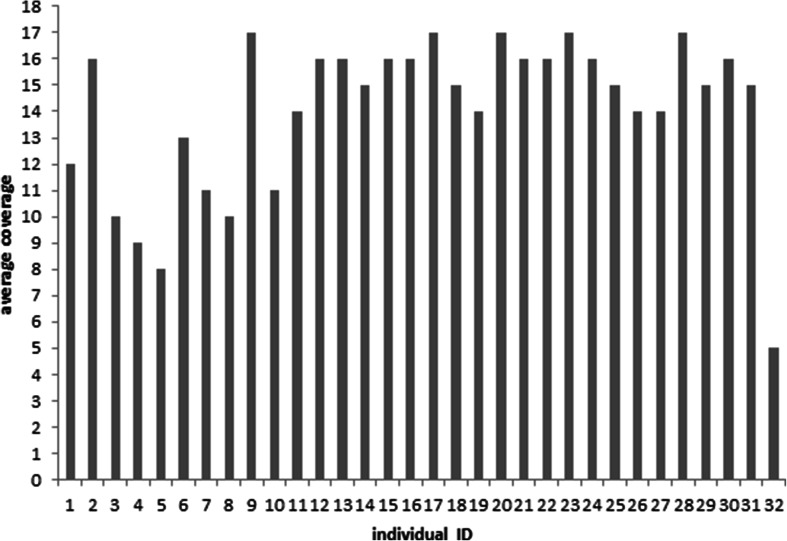
The genome averaged sequencing coverage for each individual

**Fig. 2:**
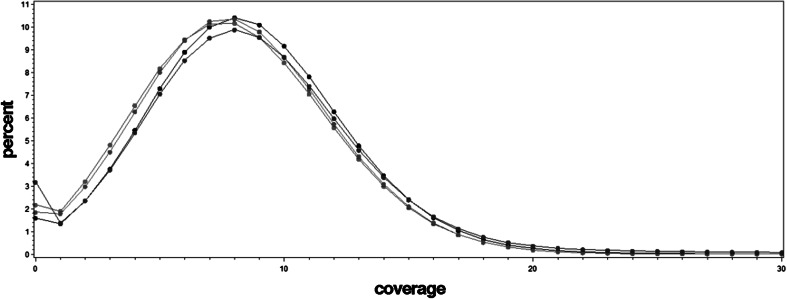
Coverage in percent observed for a particular individual on BTA1 (*black*), BTA10 (*red*), BTA20 (*green*), and BTX (*blue*) (Color figure online)

The total number of raw reads generated for a single animal varied between 164,984,147 and 472,265,620. Raw reads generated were filtered and trimmed using trimmomatic (Bolger et al. 2014) to remove low-quality base calls and sequencing adapters. Filtered reads were aligned to the UMD3.1 reference genome using BWA–MEM (Li and Durbin 2010). The resulting number of aligned reads varied from 155,202,885 to 454,412,859, which covered between 94.07 and 99.57 % of the genome, respectively. The corresponding coverage, averaged along the genome, was 14.03, ranging from 5 to 17 (Fig. 1). The coverage also varied considerably along an individual genome (Fig. 2), with some regions not covered at all and other regions with very high coverage. BAM files were processed to mark and remove duplicated reads using Picard (http://broadinstitute.github.io/picard/). Variant calling was done using the latest version of FreeBayes with filters requiring a minimum alignment quality of 30 and a minimum base quality of 20 for allele calling.

### Selected genes

Table 1 lists genes that were selected for inter-individual comparison of mononucleotide variability. The primary selection criterion was gene function for which three categories were considered: (1) housekeeping—representing genes of primary importance for the organism metabolism, and in our study genes from the commercial bovine housekeeping gene array from QIAGEN (RT^2^ Profiler™ PCR array cow housekeeping genes) were considered; (2) neutral to selection—representing genes which are supposed to remain relatively neutral to selection for modern dairy cattle according to ISAG–FAO recommendations; and (3) strongly selected—representing genes with documented strong effect on production traits in dairy cattle which are therefore supposed to be under strong unidirectional selection pressure over many generations. For calculating SNP number and density, exon regions (including UTR) within genes were considered, while introns were skipped, so that SNP density was expressed as the number of SNPs divided by the total exon length of a gene.

**Table 1:** Genes selected for comparison

Gene			BTA	SNPs	
NCBI ID	Acronym	Name		All	Missense
Housekeeping					
280979	ACTB	Actin, beta	25	110	32
280729	B2M	Beta-2-microglobulin	10	1039	0
281181	G3PDH	Glyceraldehyde-3-phosphate dehydrogenase	5	403	1
515614	HMBS	Hydroxymethylbilane synthase	15	49	0
767874	HSP90AB1	Heat shock 90 kDa protein 1, beta	23	94	32
444874	UBC	Ubiquitin C	17	431	5
Strong selection					
767906	ARL4A	ADP-ribosylation factor-like 4A	4	57	1
407216	BMP4	Bone morphogenetic protein 4	10	122	0
282609	DGAT1	Diacylglycerol *O*-acyltransferase 1	14	113	1
535043	ITGA6	integrin, alpha 6	2	3647	21
444881	MYD88	Myeloid differentiation primary response 88	22	176	0
Neutral to selection					
534958	AGTPBP1	ATP/GTP binding protein 1	8	12,176	67
520250	ANKRD32	Ankyrin repeat domain 32	7	1672	29
533894	LRP1	Low-density lipoprotein receptor-related protein 1	5	1327	11
540504	SYNE2	Spectrin repeat-containing nuclear	10	21,384	245
515119	URI1	URI1, prefoldin-like chaperone	18	3591	20

### Hypothesis testing

In order to test hypotheses regarding the inter-individual variability in SNP/indel numbers (*N*_i_) defined as $H_{0} (\forall i,i^{\prime } :N_{i} = N_{{i{\prime }}} )$ and $H_{1} (\exists i,i:N_{i} \ne N_{i\prime } )$, the Pearson’s *χ*^2^ statistics were used: $\chi^{2} = \sum\nolimits_{i = 1}^{32} {\frac{{(N_{i} - \overline{N} )}}{{\overline{N} }}} \sim \chi_{31}^{2}$, with $\overline{N}$ denoting the number of variants averaged over all individuals. Furthermore, to test differences in SNP density between chromosomes, as well as between selected genes and gene categories, a one-way and a nested two-way analyses of variance were applied with the subsequent *F* tests for overall variability and post hoc *t* tests for assessing the differences between particular groups.

## Results and discussion

### Single-nucleotide polymorphisms

The total number of SNPs identified per individual ranged between 2,063,811 and 6,117,976, with a standard deviation of 663,223, which accounts for between 0.08 and 0.23 % of the total genome length. A cow with a notably low number of polymorphisms was excluded from further comparisons involving individual variability of variants. Still, for the remaining animals, the differences in the number of detected SNPs were highly significant (*P* < 10^−20^). Similar numbers of SNPs were reported by Jansen et al. (2013) ranging between 5,885,050 and 6,366,501 SNPs, by Baes et al. (2014) ranging between 5,854,886 and 6,404,094, by Kõks et al. (2013)—5,932,230 SNPs for a single sequenced cow, and by Kõks et al. (2014)—6,362,988 SNPs for a single sequenced bull, whereas a lower number of 3,755,633 SNPs was reported for a single bull by Stothard et al. (2011). Short sequences from all of the above individuals were aligned to the same (UMD3.1) reference genome as was the case in our study, but depending on the study, represented various breeds (Fleckvieh, Jersey, Swiss dairy cattle, or Holstein–Friesian).

**Fig. 3:**
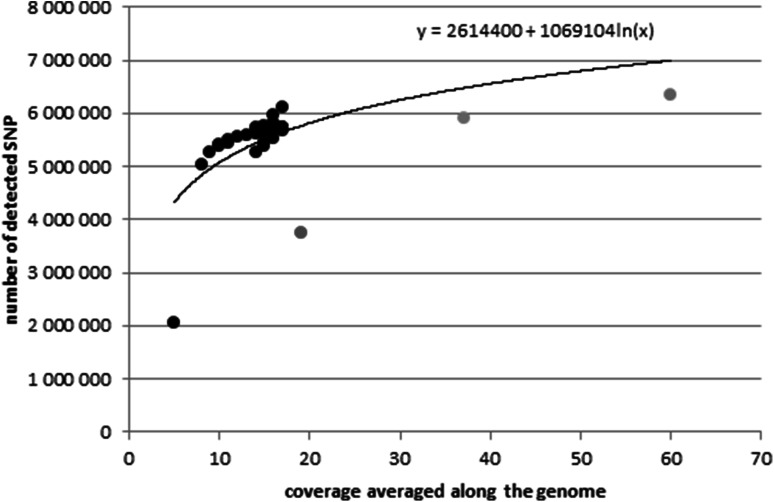
The total number of detected SNPs and related coverage for data representing our study (*black*) enhanced by data from Stothard et al. (2011) in *red*, Kõks et al. (2013) in *blue*, and Kõks et al. (2014) in *green*. The best fitting function is superimposed on the data points (Color figure online)

By looking at the numbers of SNPs identified per individual in our sample and the corresponding average genome coverage presented in Fig. 3, it is evident that the two quantities are related. In order to quantify this relationship, various regression models including a linear regression, a second-grade polynomial, and a logarithmic model were fitted to the data available in our study complemented by the data from Kõks et al. (2013, 2014) and Stothard et al. (2011), which provided information on the number of SNPs obtained by high coverage. The logarithmic model fitted the best (Fig. 3) indicating a non-linear relationship between the number of detected SNPs and the genome coverage. In particular, a higher increase in SNP number was observed for the low coverage situation, rather than in higher coverage genomes. For instance, a rise in coverage from 5× to 10× is expected to increase the number of detected SNPs by 741,046, whereas a rise in coverage from 60× to 65× results in an increase in the number of identified SNPs by only 85,574. However, it should be noted that due to (1) the fact that data compiled from different studies were analyzed together, (2) a relatively small number of observations was available for high coverage data, and (3) a series of potentially important variables, such as sequencing technology, variation in read depth along the genome, alignment and variant calling methodology, individual level of genomic variation which is a function of inbreeding coefficient, as well as the level of genetic relatedness to the donor of the reference genome, and SNP filtering criteria were neglected, and these comparisons only provide a rough description of the relationship between average genome coverage and the number for polymorphisms detected.

**Fig. 4:**
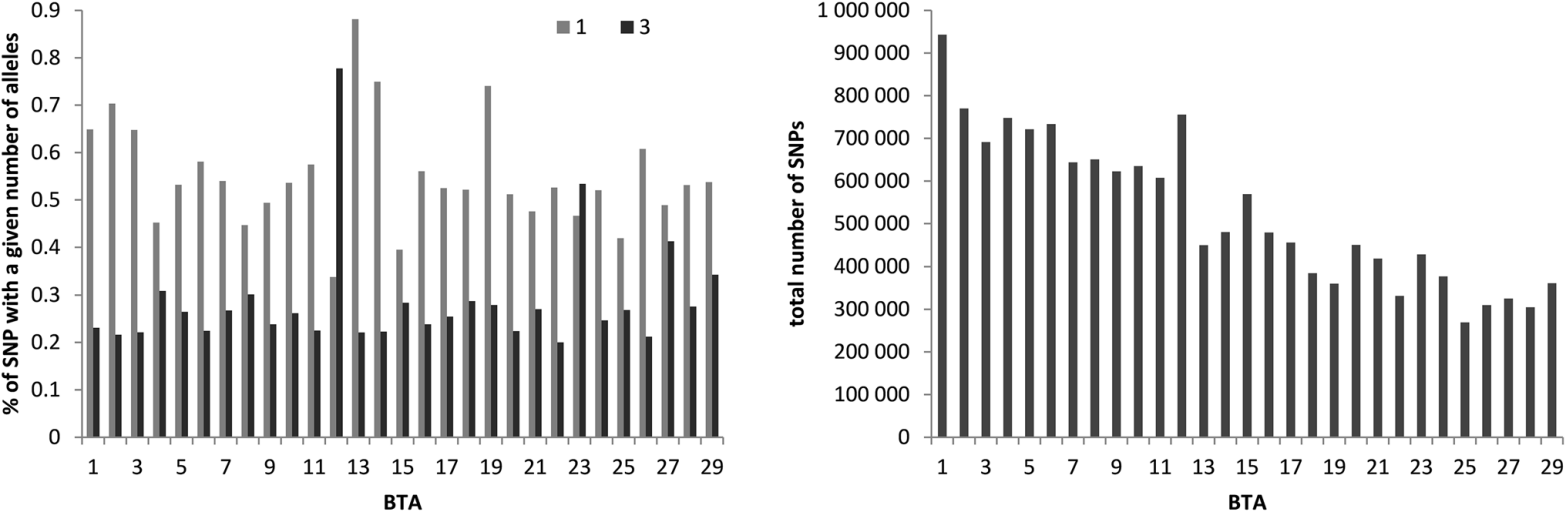
The percentage of SNPs with *1* and* 3* alleles as well as the total number of SNPs identified across 32 individuals

A total of 15,272,618 SNPs were identified across all the 32 individuals, and among them, 575,215 SNPs were common for all sequenced animals (excluding the cow with exceptionally low coverage). The vast majority of SNPs were bi-allelic (99.159 %). However, there were also monomorphic (i.e. only alternative homozygous individuals) SNPs (0.551 %), tri-allelic SNPs (0.289 %), and even a small fraction of four-allelic SNPs (0.001 %) (Fig. 4). Although rare, such SNPs need special attention in further processing of data since they may arise from sequencing, alignment errors, or strong selection (monomorphic SNPs) but may also represent genomic sites of high mutation rate in the bovine genome (tri- and four-allelic SNPs).

### Genomic distribution of missense SNPs

We considered the distribution of SNPs identified among the 32 cows within coding regions, comparing genes belonging to three different categories: housekeeping genes (HK), genes undergoing strong selection in dairy cattle (SS), and genes neutral to selection (NS). Significant differences in SNP density for missense and stop codon removing SNPs were observed between gene categories and between individual genes, with the density for NS being the highest with 1.235 SNPs per Mbp, density of 0.261 SNP per Mbp being the intermediate for HK, and density of 0.059 SNP per Mbp for SS being the lowest. Also the raw number of missense SNPs was significantly higher within NS genes (2.33 SNPs per gene on average) than within HK (0.36 SNP) and SS (0.14 SNP) categories. The low number and density of SNPs identified within HK and SS was expected from the evolutionary perspective, both regarding natural and artificial selection, since potentially protein changing mutations have metabolic consequences ranging from a benign effect (58 % of nonsynonymous SNPs) to a damaging effect (24 % of nonsynonymous SNPs)—as predicted by Jansen et al. (2013).

Additionally, we considered polymorphisms located within the 3′-UTR and 5′-UTR regions marking the parts of genes that possibly affect the expression of exons. It was found that both their number and density varied significantly between gene categories as well as between genes. In particular, a significant difference in density was observed between the HK group with 0.621 SNPs per Mbp and the other two categories—NS with 1.491 SNPs and SS with 1.572 SNPs per Mbp. The highest average number of UTR SNPs per gene was equal to one for SS genes, followed by 0.34 for NS and 0.23 for HK, which is in contrast to the number of SNPs in coding regions of these genes. The discrepancy might at first glance seem surprising, but Larizza et al. (2002) found that the evolutionary dynamics of the UTRs is rather different from that of coding regions. This results from differences in functional constraints: 5′ and 3′-UTRs that bracket coding sequences are fundamental structural and regulatory regions of eukaryotic genes (Larizza et al. 2002; Mignone et al. 2002; Ptashne and Gann 2001; Wilkie et al. 2003). As demonstrated by Larizza et al. (2002), UTRs are much more divergent in terms of length and sequence across species of mammals (*Homo sapiens*, *Bos taurus*, *Mus* sp) than the corresponding coding regions. This is, among other reasons, due to the presence of repetitive elements in the UTRs, which are not found in coding regions.

### Technical error in SNP detection

**Fig. 5:**
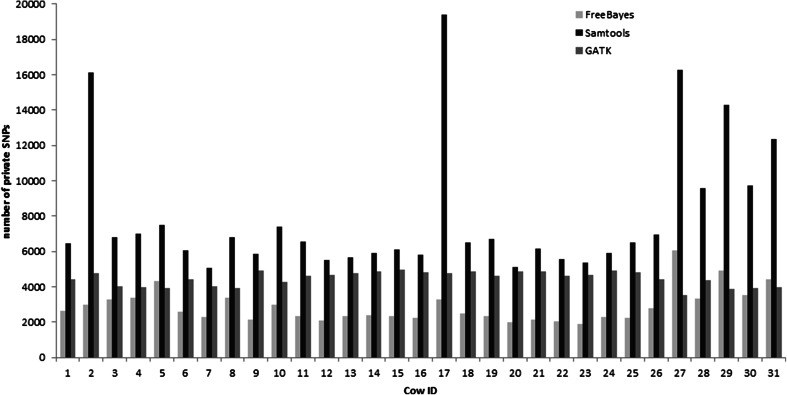
The number of private SNPs identified on BTA10 using FreeBayes, Samtools, and GATK

Using BTA10 as an example chromosome we evaluated differences in the number of SNPs identified by bioinformatic pipelines differing only in the applied variant calling software—FreeBayes (http://arxiv.org/abs/1207.3907), GATK (McKenna et al. 2010), or Samtools (Li et al. 2009). Considering the output of FreeBayes as a baseline only 93.5 % of SNPs were common between pipelines. A clear pattern in the number of “private” SNPs, i.e., SNPs which that identified only by one of the applied software packages, was observed across animals (Fig. 5). For all cows, Samtools detected the highest number of private polymorphisms, whereas for 29 out of 31 cows, it was FreeBayes, which resulted in the lowest number of private SNPs. A *t* test comparison of the Phred scaled probabilities associated with all common and private SNPs identified on BTA10 across all cows revealed that for FreeBayes and Samtools, the SNP likelihood was significantly lower for privately called SNPs. The opposite was observed for GATK. Our results do not correspond to the study of Baes et al. (2014) who identified a higher total number of SNPs using GATK than Samtools.

### Oligonucleotide insertions and deletions

The number of insertions and deletions identified in the genomes of the cows under study was much lower than the number of SNPs identified. Indels covered approximately 0.02 % of the genome, with numbers ranging from 261,712 to 330,103 and from 271,398 to 343,649 for insertions and deletions, respectively. This was very similar to the number of indel polymorphisms reported by Baes et al. (2014) remaining within the range of 496,203–832,689 per animal, depending on the applied variant detection pipeline.

The number of insertions and deletions differed significantly among cows (*P* < 10^−20^ in both cases), but a very high significant correlation of 0.996 was observed between the numbers of insertions and deletions identified for each animal. Correlations between the number of identified SNPs and mononucleotide deletions/insertions were somewhat lower, but also significant, amounting to 0.792 and 0.747, respectively.

## Conclusions

Nowadays, datasets allowing for the population-wide conclusions are available not only for humans but also for other species. Thanks to very detailed records of binary (e.g. disease) and quantitative phenotypes, as well as environmental factors underlying phenotype expression, very deep pedigrees, and the relative ease of obtaining data from large groups of animals of predefined familial relationship structure, domestic cattle can play a role not only as production animals but also as model organisms. Using a dataset from 32 individuals, we were able to conduct an analysis of the genome mononucleotide variation in *Bos taurus* based on formal hypothesis testing.

The importance of statistically supported analysis can be best visualized by comparing results for whole-genome sequence variation of a single Holstein cow (Kõks et al. 2013) and a single Holstein bull (Kõks et al. 2014) obtained with the same sequencing methodology and bioinformatics procedures. There is a low repeatability of results reported in both studies that arises due to sampling error. For instance, the lists of 58 genes with the largest and smallest numbers of SNPs presented in both abovementioned studies contains only one overlap between a male and a female, which is within the range of 5 % type I error.

Based on whole-genome sequences of 32 cows, we were able to demonstrate statistically significant inter-individual variation in the number of SNPs and indels. An important issue in variant-based analysis of next-generation sequencing (NGS) data has always been a high level of technological error underlying variant identification (Baes et al. 2014; Lee et al. 2013). Still, we believe that due to our use of the same (1) data preparation procedure involving DNA extraction and sequencing, (2) variant detection pipeline for all individuals, and (3) incorporation of statistical analysis, our results remain valid. Note that the estimated variation in the number of SNP, insertion, and deletion sites in the animals under study expresses the degree of deviations from the UMD3.1 reference sequence used in this study. Hence, the higher or lower number of identified variants in individual cows may be due to the specificity of the applied reference genome [as demonstrated for humans by Li (2014)], which originates from the Hereford breed and thus does not reflect the genetic variation representative of the Holstein–Friesian population.

Moreover, we also observed significant variation on a functional basis, by comparing the numbers and density of SNPs identified within genes representing different functional categories. The highest rate of SNPs within genes being neutral to selection in dairy cattle supports the hypothesis that natural selection tends to eliminate variation within housekeeping genes, whereas artificial selection for milk production pre-imposed on dairy cattle for many generations suppresses variation within genes responsible for the selected trait. We observed that the sequences of UTR appeared to be more variable than those of exons, which empirically proves the hypothesis that the predominant type of selection in non-coding sequences is a positive one, i.e., favoring sequence diversity, whereas in coding sequences, there is a very strong negative selection pressure that leads to elimination of new mutated alleles. Moreover, the most variable UTR sequence was observed in genes undergoing strong selection, which is in agreement with Hong et al. (2006). This phenomenon can be related to the possible function of those regions in the regulation of translation, which can be important for highly expressed genes of individuals characterized by a very high milk production level.
